# Dataset on the associations between sleep quality/duration and cognitive performance in cognitively healthy older adults

**DOI:** 10.1016/j.dib.2017.08.028

**Published:** 2017-09-01

**Authors:** A. Tsapanou, Y. Gu, D.M. O'Shea, M. Yannakoulia, M.H. Kosmidis, E. Dardiotis, G. Hadjigeorgiou, P. Sakka, Y. Stern, N. Scarmeas

**Affiliations:** a Department of Social Medicine, Psychiatry and Neurology, National and Kapodistrian University of Athens, Athens, Greece; b Cognitive Neuroscience Division, Department of Neurology and The Taub Institute for Research on Alzheimer's Disease and the Aging Brain, Columbia University Medical Center, New York, NY, USA; c Department of Clinical and Health Psychology, University of Florida, FL, USA; d Department of Nutrition and Diabetics, Harokopio University, Athens, Greece; e Department of Psychology, Aristotle University of Thessaloniki, Greece; f Department of Neurology, Faculty of Medicine, University of Thessaly, Larissa, Greece; g Athens Association of Alzheimer's Disease and Related Disorders, Athens, Greece

**Keywords:** HELIAD, Hellenic Longitudinal Investigation of Aging and Diet, *ApoE*-ε4, *Apolipoprotein E-ε4*, MCI, Mild Cognitive Impairment, AD, Alzheimer's Disease, MOS, Medical Outcomes Study, Sleep duration, Sleep quality, Aging, Cognition

## Abstract

In the present data, we provide the details of the cross-sectional study examining the associations between sleep quality/sleep duration and cognitive performance. Data are from the Hellenic Longitudinal Investigation of Aging and Diet (HELIAD). A total of 1484 older adults (65 y.o. or older) took part in the study. Sleep measurements were drawn from the sleep scale of the Medical Outcomes Study (MOS). Cognition was used as a z-score drawn by different tests. The domains examined were: executive function, visuo-spatial ability, language, attention- speed of processing, as well as the composite z-score of all the cognitive domains (including memory). Linear regression models were conducted to investigate the associations between sleep quality and cognition, and sleep duration and cognition as well. We also conducted linear regression analyses for the associations between sleep quality/duration and cognitive domains/composite cognitive score based on the status of the *Apolipoprotein E-ε4* (*ApoE*-ε4) genotype. Analyses were performed excluding both the demented and the Mild Cognitive Impairment (MCI) participants. Adjustments conducted for multiple covariates. For further analyses and enhanced discussion, see original article: “Sleep quality and duration in relation to memory in the elderly: initial results from the Hellenic Longitudinal Investigation of Aging and Diet” by Tsapanou et al. [1]

**Specifications Table**

**Table:** 

Subject area	Neuropsychology, Neurology
More specific subject area	Clinical Neuropsychology
Type of data	Tables, Graphs, Questionnaire
How data was acquired	Quantitative data of 1589 elderly, using a self-reported sleep questionnaire, and cognitive tests as well
Data format	Raw data, analyzed
Experimental factors	Sleep quality, sleep duration, and memory performance were the main variables used. Age, sex, education, and body mass index were used as covariates. Clinical co-morbidities were used as further covariates
Experimental features	Characterization of the sleep status
Data source location	Athens, Greece
Data accessibility	Data in this article

**Value of the data**

• The data can help Identifying the sleep measures associated with specific cognitive domains.
• Providing data about cognitive sub-categories could be comparable to other studies and help developing specific training programs for the improvement of the older adult's cognition.
• Providing data for the associations between sleep and cognition in different *ApoE*-ε4 status can help other researchers examine other genotypes as well.
• This data allows other researchers to extend the statistical analyses on longitudinal design studies.

## 1 Data

The dataset of this article provides information about the cross-sectional study that examined the association between sleep quality/duration and specific cognitive domains other than memory, in a large group of older adults. We also conducted the above analyses based on the *ApoE*-ε4 genotype. We provide the results of the different cognitive groups as well as of the composite cognitive score.

## 2 Experimental design, materials and methods

### 2.1 Sample collection

Participants were drawn from the HELIAD study. HELIAD is a population-based, multidisciplinary, collaborative study designed to estimate the prevalence and incidence of Mild Cognitive Impairment (MCI), Alzheimer's Disease (AD), other types of dementia, as well as other neuropsychiatric conditions of aging in the Greek population. The study includes several demographic, medical, social, environmental, clinical, nutritional, and neuropsychological determinants as well as the lifestyle activities of each participant. All participants were 65 y.o. or older [1].

### 2.2 Sleep measures

Sleep quality was assessed using the Sleep Scale from the Medical Outcomes Study (MOS). This scale is a self-reported 12-item questionnaire [2]. Based on the manual of the specific scale [3], in the current study, sleep quality was examined by the Sleep Index II (see Appendix).

In order to also examine sleep duration, we used the following question: “On the average, how many hours did you sleep each night during the past 4 weeks? Write in number of hours and minutes per night.” The final variable used was the sum of the total duration calculated in minutes.

### 2.3 Analysis

We used general linear models (GLM) with the sleep quality first and then the sleep duration a variable as the predictor, and the different cognitive z-scores as the outcome.

In the model we adjusted for age, sex, education, and sleep medication (Table 1, Table 2, Table 3, Table 4).

**Table 1:** Association between sleep quality and cognitive domains/composite z-score in the non-demented, non-MCI group. Adjusted for age, sex, education, and sleep medication.

**Cognitive domain**	***β***	***p***
**Executive**	−0.004	0.837
**Visuo-spatial**	−0.045	0.057
**Language**	−0.053	**0.006**
**Attention- speed**	0.001	0.974
**Composite**	−0.036	0.056

**Table 2:** Association between sleep duration and cognitive domains/composite z-score in the non-demented, non-MCI group. Adjusted for age, sex, education, and sleep medication.

**Cognitive domain**	***β***	***p***
**Executive**	−0.055	**0.008**
**Visuo-spatial**	0.030	0.197
**Language**	−0.015	0.418
**Attention- speed**	−0.014	0.549
**Composite**	−0.046	**0.014**

**Table 3:** Association between sleep quality and cognitive domains/composite cognitive score in the carriers and non-carriers of the ApoE-ε4. Analyses on the non-demented, non-MCI sample, adjusted for age, sex, education, and sleep medication.

**Cognitive domain**	***ApoE-ε4*****carriers**	***ApoE-ε4*****non-carriers**
	***β*,*****p***	***β*,*****p***
**Executive**	−0.020, 0.756	0.031, 0.291
**Visuo-spatial**	−0.011, 0.848	−0.025, 0.445
**Language**	−0.112, 0.067	−0.006, 0.814
**Attention- speed**	0.097, 0.178	0.014, 0.669
**Composite**	−0.007, 0.800	−0.007, 0.800

**Table 4:** Associations between sleep duration and cognitive domains/composite cognitive score in the carriers and non-carriers of the ApoE-ε4. Analyses on the non-demented, non-MCI sample, adjusted for age, sex, education, and sleep medication.

**Cognitive domain**	***ApoE-ε4*****carriers**	***ApoE-ε4*****non-carriers**
	***β*,*****p***	***β*,*****p***
**Executive**	0.096, 0.132	−**0.110, ≤0.0001**
**Visuo-spatial**	0.048, 0.489	−0.046, 0.157
**Language**	**0.134, 0.025**	−**0.061, 0.020**
**Attention- speed**	−0.078, 0.276	−0.028, 0.389
**Composite**	0.048, 0.373	−**0.085, 0.001**

## Funding sources

This study was supported by the following grants: IIRG-09-133014 from the Alzheimer's Association; 189 10276/8/9/2011 from the ESPA-EU program Excellence Grant (ARISTEIA), which is co-funded by the European Social Fund and Greek National resources, ΔΥ2β/οικ.51657/14.4.2009 from the Ministry for Health and Social Solidarity (Greece), ‘Research Excellence Programs IKY/Siemens’ fellowship

## Footnotes

Appendix A Supplementary data associated with this article can be found in the online version at doi:10.1016/j.dib.2017.08.028.
