# Supplementary material for: Dataset on the associations between sleep quality/duration and cognitive performance in cognitively healthy older adults
Source: Data Brief. 2017 Sep 1;14:720–3. doi: 10.1016/j.dib.2017.08.028 (PMC5596325; doi:10.1016/j.dib.2017.08.028)
Supplement: Supplementary file 2 — Supplementary material [file mmc2.docx]

# Appendix

Sleep Scale from the Medical Outcomes Study

1. How long did it usually take for you to fall asleep during the past 4 weeks?

(Circle One)

0–15 minutes…………..….1

16–30 minutes…………….2

31–45 minutes…………….3

46–60 minutes…………….4

More than 60 minutes …….5

2. On the average, how long did you sleep each night during the past 4 weeks?

Write in number of hours per night:

How often during the past 4 weeks did you:

3. Feel that your sleep was not quiet (moving restlessly, feeling tense, speaking, etc., while sleeping)?

4. Get enough sleep to feel rested upon waking in the morning?

5. Awake short of breath or with a headache?

6. Feel drowsy or sleepy during the day?

7. Have trouble falling asleep?

8. Awake during your sleep time and have trouble falling asleep again?

9. Have trouble staying awake during the day?

10. Snore during your sleep?

11. Take naps (5 minutes or longer) during the day?

12. Get the amount of sleep you needed?

Possible answers: 1=All of the time, 2=Most of the time, 3=A good bit of the time, 4=Some of the time, 5=A little of the time, 6=None of the time

Hays, R. D., & Stewart, A. L. (1992). Sleep measures. In A. L. Stewart & J. E. Ware (eds.). Measuring functioning and well-being: The Medical Outcomes Study approach (pp. 235–259), Durham, NC: Duke University Press.

Hays RD, Martin SA, Sesti AM, Spritzer KL. Psychometric properties of the Medical Outcomes Study Sleep measure. Sleep Med. 2005;6(1):41-4.
